# Identification of Driver Status Hazard Level and the System

**DOI:** 10.3390/s23177536

**Published:** 2023-08-30

**Authors:** Jiayuan Gong, Shiwei Zhou, Wenbo Ren

**Affiliations:** 1 Harbin Engineering University, Harbin 150001, China; 20160013@huat.edu.cn; 2 Institute of Automotive Engineers, Hubei University of Auomotive Technology, Shiyan 442002, China; 202111205@huat.edu.cn; 3 Shiyan Industry Technique Academy of Chinese Academy of Engineering, Shiyan 442002, China

**Keywords:** YOLOX, driver danger levels, Dlib, Image Identification

## Abstract

According to the survey statistics, most traffic accidents are caused by the driver’s behavior and status irregularities. Because there is no multi-level dangerous state grading system at home and abroad, this paper proposes a complex state grading system for real-time detection and dynamic tracking of the driver’s state. The system uses OpenMV as the acquisition camera combined with the cradle head tracking system to collect the driver’s current driving image in real-time dynamically, combines the YOLOX algorithm with the OpenPose algorithm to judge the driver’s dangerous driving behavior by detecting unsafe objects in the cab and the driver’s posture, and combines the improved Retinaface face detection algorithm with the Dlib feature-point algorithm to discriminate the fatigue driving state of the driver. The experimental results show that the accuracy of the three driver danger levels (R1, R2, and R3) obtained by the proposed system reaches 95.8%, 94.5%, and 96.3%, respectively. The experimental results of this system have a specific practical significance in driver-distracted driving warnings.

## 1. Introduction

With the progress of science and technology and the improvement of people’s living standards, the number of automobile owners and drivers is increasing rapidly. According to the Traffic Management Bureau of the Ministry of Public Security report, there have been more than 200,000 traffic accidents in China every year in the past ten years, with more than 260,000 casualties and CNY 1.2 billion of direct economic losses [1,2,3]. Among them, the driver is an essential factor affecting driving safety and road smoothness, and it is necessary to analyze and pay attention to the driver’s behavior [4,5,6]. Some research data from developed countries in Europe and the United States show that the proportion of traffic accidents caused by human factors is as high as 80% to 90% [7,8,9]. Therefore, it is of great significance to study a system for discriminating the degree of danger of a driver’s state [10,11].

Hong S, Kwon H et al. [12] combined conventional photoelectric volumetric tracing (PPG) and electrocardiographic tracing (ECG) in the ear canal and found that fatigue detection was highly accurate. Sivaraman S, Trivedi M M et al. [13] at the University of California analyzed driving status by determining the vehicle’s position concerning the rest of the vehicles through video images. The Face LAB system [14], created by the Australian National University in collaboration with Volvo, first recognizes the driver’s facial features. It obtains data such as blinking frequency, pupil diameter, degree of eyelid closure, head position and rotation frequency, and mouth and eyebrow movement parameters, which are fused together to analyze and obtain the driver’s state.

The physiological parameter detection method is subjective; the vehicle behavior detection method is affected by the road; and the machine vision detection method is costly. It is impossible to prejudge dangerous behavior, and the detection effect of a single judgment standard is poor. Therefore, this paper proposes a comprehensive driver hazardous state behavior discrimination system, which combines the YOLOX target detection algorithm and OpenPose driver gesture recognition algorithm to detect the driver’s hazardous behavior. They are combined with driver dynamic fatigue tracking detection, driver unsafe state, and the behavior hazard classification detection system [15,16,17,18,19]. The system sends real-time warnings to the driver according to the degree of danger so that the driver can adjust the state and behavior in time to ensure the safety of driving, thus improving the driving safety coefficient and reducing the occurrence of traffic accidents.

## 2. Object Recognition in YOLOX Cab

The target detection problem is one of the more difficult problems in machine vision due to the significant disparity in the target object’s shape and the influence of light intensity, occlusion, and other factors in the detection process. Deep learning-based target detection algorithms are divided into two categories: two stage and one stage. Two-stage algorithms first generate a series of candidate frames before using a convolutional neural network to classify the target, such as R-CNN, SPP-Net, Fast R-CNN, Faster R-CNN, and R-FCN, etc. Whereas, one-stage algorithms extract features directly from the network to predict object classification and location, e.g., YOLOv1, YOLOv2, RetinaNet, YOLOv3, and SSD. Although the YOLO algorithm is less effective in the detection of small target objects, since the targets in this paper, cigarettes, mugs, and phones in the cab, i.e., the images throughout the detection process, are not small objects, and also the YOLOX detection rate is speedy, the enhanced version can run at 45fps (frames per second) on the GPU, and the simplified version can even reach 155fps. In addition, YOLOX has good generalization ability and can correlate well with the background information when recognizing objects. Therefore, the algorithm satisfies the most needed real-time in this study and can significantly reduce the false detection of targets in the cab.

**Figure 1:**
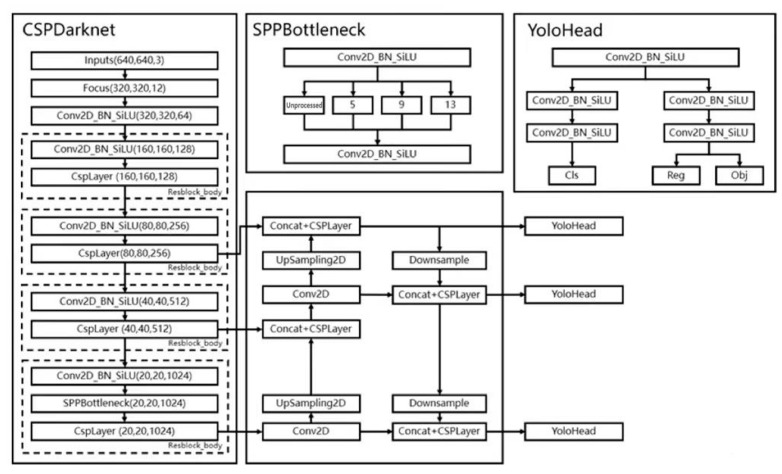
YOLOX network structure.

YOLOX can be divided into three parts: CSPDarknet, FPN, and YOLO Head, and the YOLOX network structure is shown in Figure 1.

The backbone feature extraction network of YOLOX is CSPDarknet; after inputting the image, the feature extraction is first carried out in the backbone network, which is called the feature layer, storing the feature set of all the input images. The backbone of YOLOX is composed of residual convolution; the residual network has the advantages of easy optimization and improvement of accuracy. After the input image, it first passes through the Focus structure, which compresses the width and height to 1/2 of the original, extends the number of channels by four times, and then passes through the Resblock_body structure four times. The three feature layers extracted are located in the middle, lower middle, and bottom layers of the CSPDarknet, respectively. When the input is (640,640,3), the shape sizes of the three feature layers are feature1 = (80,80,256), feature2 = (40,40,512), and feature3 = (20,20,1024). The three feature layers are then fed into the FPN layer, the feature pyramid that fuses different types of shape feature layers for better feature extraction. Also, the structure of PANet is used in YOLOX to obtain the final effective feature layer by upsampling and downsampling the features.

YOLO Head is the classifier and regressor of YOLOX, and the three enhanced adequate feature layers obtained are fed into YOLO Head through the CSPDarknet and FPN network structure, each of which has width, height, and number of channels with standard convolution and activation functions. The prediction consists of three parts: Reg, Obj, and Cls. The Reg part is the regression parameter judgment of the feature points; the Obj part is the judgment of whether the feature points contain objects; and the Cls part is the kind of objects contained in the feature points.

**Figure 2:**
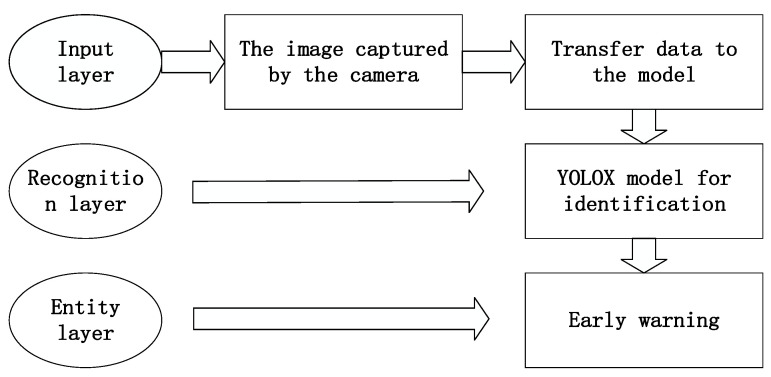
Target detection system framework.

Figure 2 shows the flow chart of the target detection system framework based on the YOLOX algorithm, which is divided into three parts: input layer, recognition layer, and entity layer.

Input layer: a camera that collects images is placed in the cab, the driver’s state image is collected in real-time through the camera, and the image is transmitted to the YOLOX model.

Recognition layer: a series of image processing and target detection are performed through the recognition layer, and the trained YOLOX model is used to recognize the target objects (cigarettes, water cups, and telephones) in the cab.

Entity layer: by analyzing the target detection results, it can provide early warning to the driver, regulate the driver’s driving behavior, and reduce accidents.

**Figure 3:**
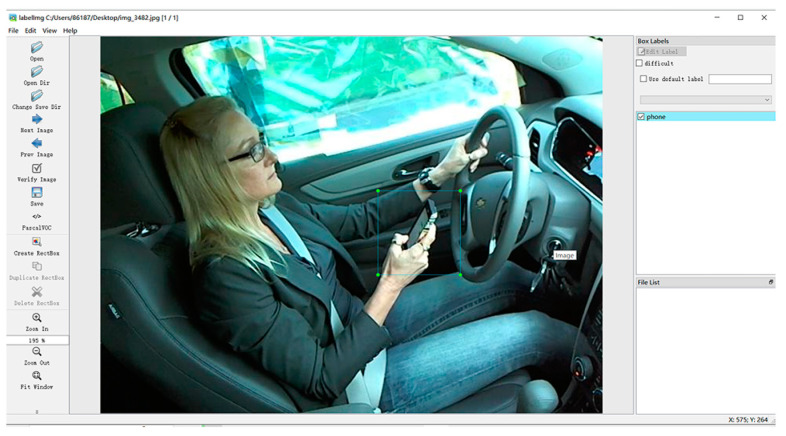
Data annotation.

No open-source dataset is available on the market for reference for the research on driver behavior and state detection. Therefore, the dataset used in this paper includes the Kaggle driving posture area image set [20,21], the collection of different drivers in different driving states. Images and some image files are downloaded from the Internet by adding noise, flipping, and increasing contrast, 6000 images are obtained, and data annotation is performed, as shown in Figure 3. The pictures are divided into three categories: smoke, drink, and phone, and a dataset is created.

**Figure 4:**
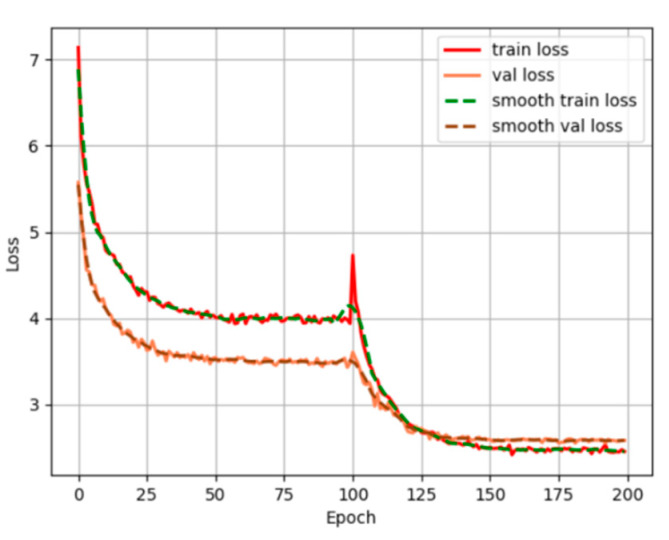
YOLOX training curve.

The YOLOX-S model is adopted, which pays more attention to the rate and meets the real-time requirements. The training process is divided into two stages: freezing and thawing. In the freezing stage, the epoch is set to 100, and the backbone of the model is frozen, which will not affect the feature extraction network and requires only a tiny amount of video memory. At the same time, freezing training helps to improve training speed, preventing weights from breaking in the initial stage. In the thawing stage, the epoch is also set to 100, the backbone of the model is thawed, and the feature extraction network changes. This stage takes up a lot of memory, and the parameters of the network change. The backbone network is first frozen and then unfrozen, the total number of training generations is set to 20, and multiple threads are started to read data to speed up the data reading process, the number of threads is set to 4, the graph is shown in Figure 4.

**Figure 5:**
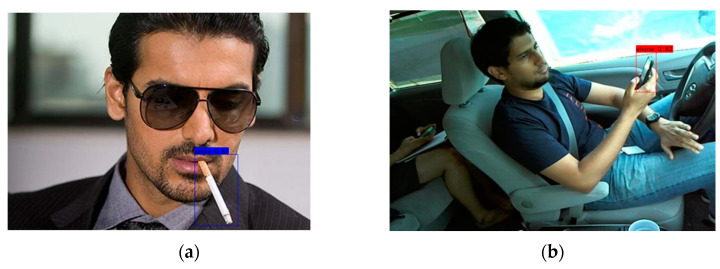
(**a**) Cigarette test results; (**b**) phone test results.

The prediction phase uses two files: yolo.py and predict.py. Modify model_path and classes_path in yolo.py. Modify model_path to the trained weight file ep200-loss2.450-val_loss2.579.pth, classes_path points to the txt file corresponding to the detection category, and predict the target image containing specific objects (water cups, cigarettes, and telephones), as shown in Figure 5.

The model precision and recall are calculated as shown in (1) and (2).

(1) $$\mathrm{Precision}=\frac{TP}{TP+FN}\times 100\%$$

(2) $$\mathrm{Recall}=\frac{TP}{TP+FP}\times 100\%$$

The test results are shown in Table 1.

From the test results, we can conclude that the accuracy rates of the three target objects, cigarettes, water cups, and telephones, are all over 95%.

**Figure 6:**
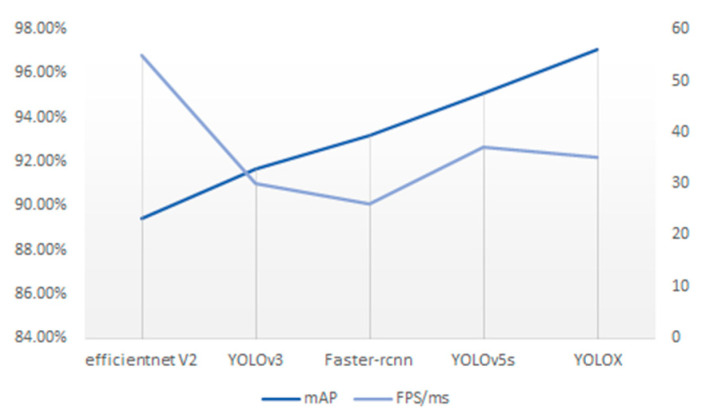
Detection efficiency of different models.

In order to verify the reasonableness and feasibility of the model, other classical target detection algorithm models are applied to this dataset, and the detection results are shown in Figure 6. Comparing the results, the YOLOX algorithm has the highest accuracy and a fast detection speed, which has obvious advantages and meets the requirements of the designed system.

## 3. OPENPOSE Driver Posture Detection

**Figure 7:**
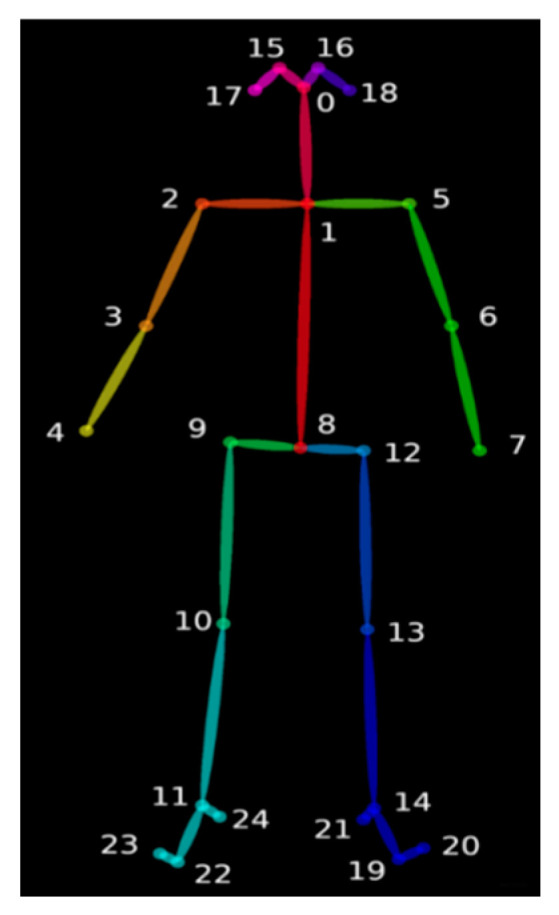
Joint point connection.

OpenPose mainly estimates the current posture of the human body through the relative position of the critical points of the human body. By observing the positions of various essential parts of the human body, the posture of the human body can be accurately measured, and the target’s posture can be predicted, such as drinking water, calling, smoking, etc. First, the posture of the driver’s body part is detected. After inputting the picture, after OpenPose processing, the bone connection points are extracted. The model used in this paper for the driver’s body is Body_25, which includes 25 human bone points. The joint point connection implementation diagram is shown in Figure 7.

**Figure 8:**
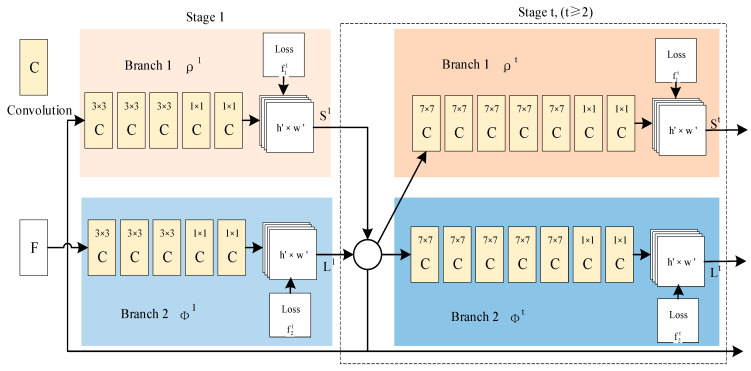
OpenPose schematic.

The OpenPose network implementation consists of the following three phases (shown in Figure 8):

Phase 1: create a feature mapping for the input image using the first ten layers of VGGNet.

Stage 2: Create a CNN network with a two-branch multi-stage. One branch predicts a set of 2D confidence maps for body part locations (e.g., eyes, elbows, knees, etc.), and the other predicts a set of 2D vector fields for partial affinities. In the first stage (left half), the network produces an initial set of detection confidence maps S and a set of partial affinity fields L. Then, in each second stage (right half), the predictions from the two branches of the previous stage are connected to the original image features F to produce more accurate predictions. This step increases the depth of the neural network to capture more accurate predictions.

Stage 3: generate 2D key points by reasoning and parsing the confidence and affinity maps through the greedy algorithm [22].

Repeating the above steps can predict the location of the key points and their confidence maps. Finally, the skeletal connectivity map of the target human body can be obtained by connecting the critical points through the greedy algorithm.

It can be seen from the detection effect that the body posture of the driver can judge whether the driver is performing behaviors that endanger driving safety, such as making a phone call, smoking, etc. Whether the driver answers the phone or not is judged by calculating the distance from the right hand to the right ear, that is, the distance from point 4 to point 17, or the distance from the left hand to the left ear, that is, the distance from point 7 to point 18. Similarly, smoking and drinking water are judged by calculating the distance from the hands to the nose, that is, the distance from points 4 and 7 to point 0.

**Figure 9:**
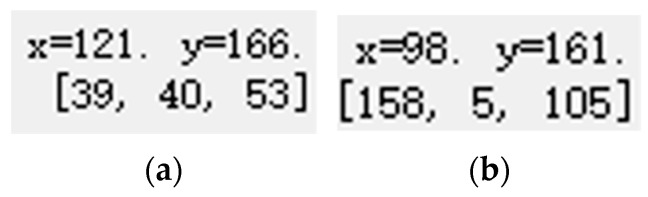
(**a**) Hand coordinates; (**b**) ear coordinates.

**Figure 10:**
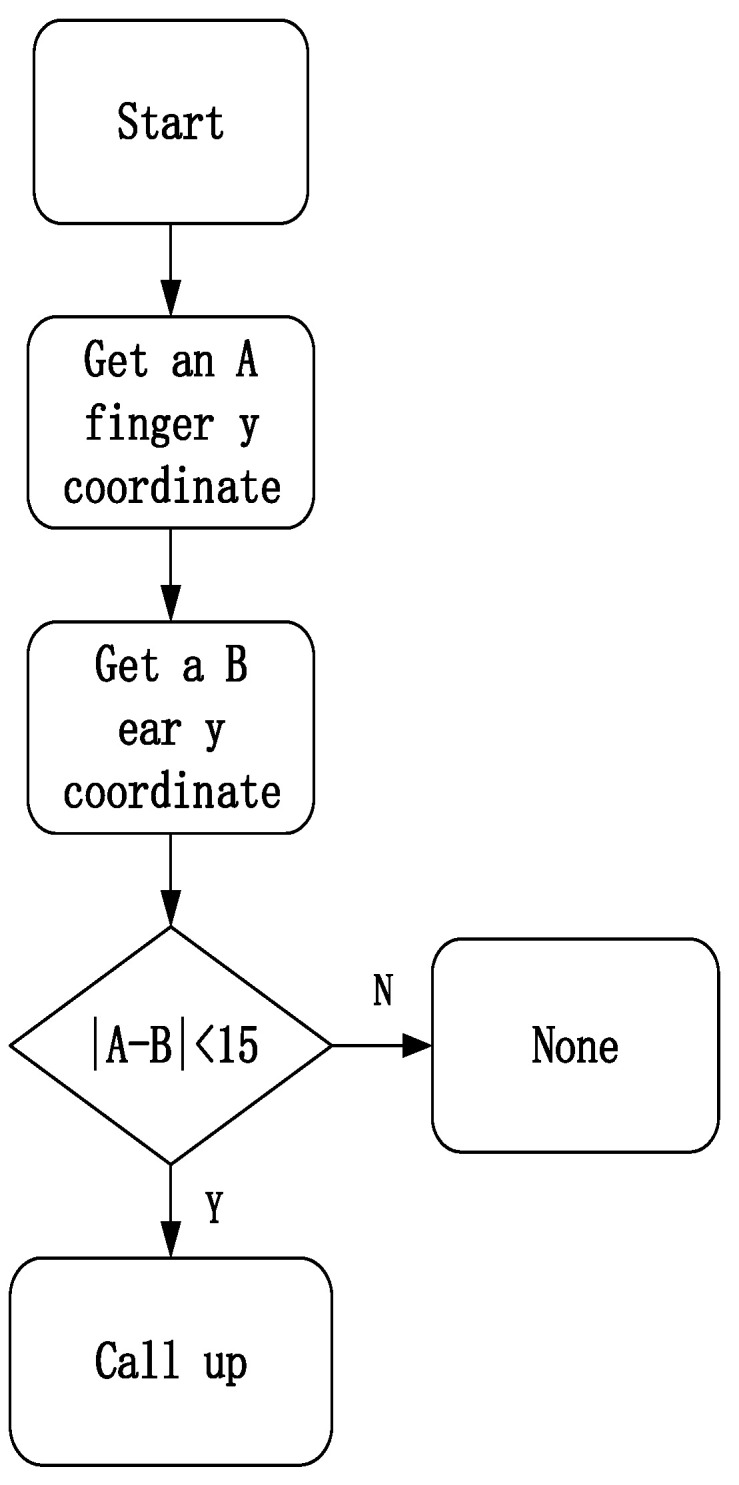
Corresponding flow chart of phone call status.

Through the coordinate value of the horizontal position of the hand key point and the horizontal position of the ear key point, that is, the *y*-axis coordinate value, it is judged whether the hand and the ear are on the same horizontal line, and thus to determine whether the driver is answering the phone (handheld phone), as shown in Figure 9. It can be seen from the common sense of making and answering calls that our hands do not coincide with the position of our ears when making calls, so this paper sets a threshold when making a judgment. If the difference between the *y*-axis coordinates of the hand and the ear is within the set threshold, it is initially considered to be receiving a call. After the assumption method and continuous experimentation, the threshold is set to 15, and the flowchart is shown in Figure 10.

As shown in Figure 9a, the *y*-axis coordinate value of the hand is 166, and as shown in Figure 9b, the *y*-axis coordinate value of the ear is 161, and the difference between them is 5, which is within the set threshold range, so it was initially judged to be on the phone.

In the same way, the driver’s smoking behavior is determined by the *y*-axis coordinate value of the hand and the *y*-axis coordinate value of the nose, and the threshold is set to 10 through the assumption method and continuous experimentation; the drinking behavior is similar to the smoking behavior. The *y*-axis coordinate value of the hand and the *y*-axis coordinate value of the nose can also be calculated for discrimination. However, the position of drinking water is more uncertain than smoking, so the threshold is set to 15.

Therefore, the driver must meet the following two conditions when smoking:

(1) The difference between the *y*-axis coordinate value of the driver’s hand joint point and the *y*-axis coordinate value of the nose joint point does not exceed 10.
(2) The YOLOX target detection system detected the presence of cigarettes.

Drivers must meet the following two conditions when drinking water:

(1) The difference between the *y*-axis coordinate value of the driver’s hand joint point and the *y*-axis coordinate value of the nose joint point does not exceed 15.
(2) There is a water cup in the detection result of the YOLOX target detection system.

The driver must meet the following two conditions when making a phone call:

(1) The difference between the *y*-axis coordinate value of the driver’s hand joint point and the *y*-axis coordinate value of the ear joint point does not exceed 15.
(2) The detection result of the YOLOX target detection system has the presence of a phone.

Since the driver’s state is continuous, it is impossible to complete these dangerous behaviors instantaneously, and the detection of the driver’s driving state image is relatively independent; there is no contextual connection, and practical information exchange cannot be performed. Some unexpected situations, such as the vehicle turning quickly, road potholes, vehicle bumps, etc., will cause sudden jumps in the captured images. The expected effect cannot be achieved in a period, so it cannot be accurately and stably obtained—test results.

Based on this, this paper introduces a voting mechanism to reduce false detections due to image jumps, enhancing the algorithm’s robustness. Based on the hardware conditions and reasonable assumptions of the camera used in this paper, a 12-frame voting queue is set up as a voter for driver behavior prediction. If, and only if, the same behavior exists in the 12-frame voting queue for more than eight frames, the output driver state will change. Otherwise, the output state will remain unchanged. Table 2 shows the comparison results of the detection accuracy with and without the voting mechanism.

The test results show that the detection accuracy of drivers drinking water has increased from 95% to 98%; the detection accuracy of smoking has increased from 93% to 95%; and the detection accuracy of phone calls has increased from 94% to 98%.

## 4. Fatigue Detection

In this paper, the driver’s face is detected based on the RetinaFace algorithm, proposed by Insight Face in 2019, incorporating excellent modeling ideas such as a feature pyramid network and enhanced feature extraction, and performs well on the WiderFace dataset [23].

**Figure 11:**
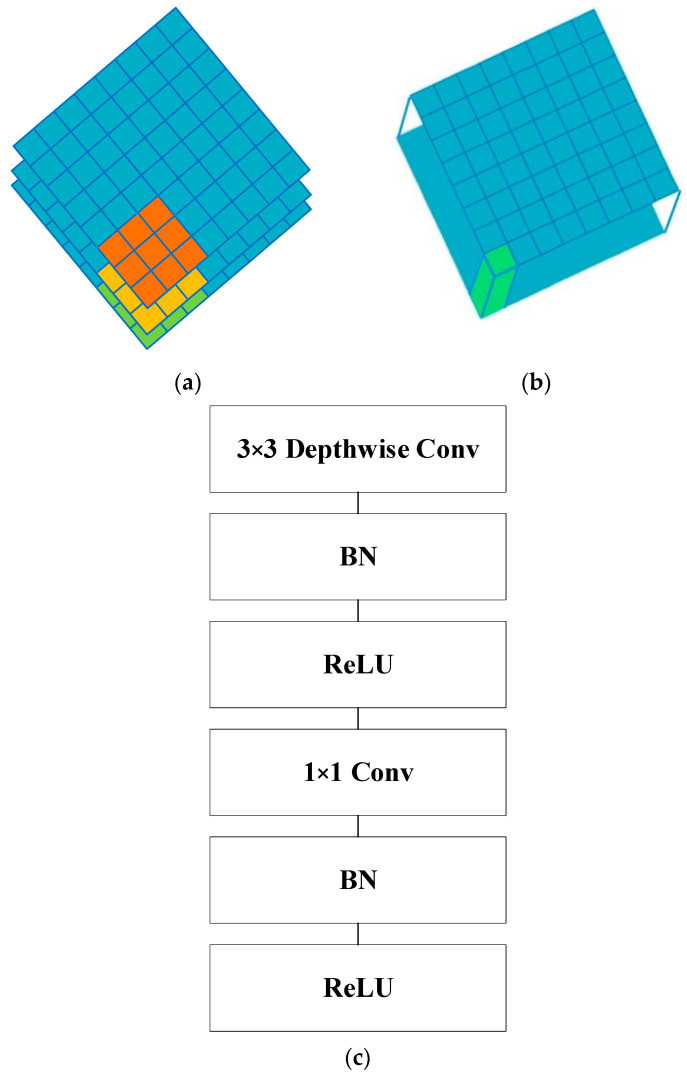
(**a**) Depthwise convolutional filters; (**b**) pointwise convolution filters; (**c**) depthwise separable convolution.

The MobileNetV1-0.25 backbone network was chosen to significantly reduce the computation and speed up the computation rate to meet the real-time nature of face detection. The MobileNet model is a lightweight deep neural network that uses the core idea of depthwise separable convolution, whose structure is shown in the following figure. Its structure is shown in Figure 11.

The prediction result of RetinaFace is divided into three, which are classification prediction result (Class Head), regression prediction result of the box (Box Head), and regression prediction result of face key points (Landmark Head), while the fatigue detection system in this paper is added with a cradle head tracking system in order to avoid affecting the accuracy of the algorithm in case of an undetectable driver’s face. Meanwhile, the Dlib algorithm is used to obtain the critical points of the driver’s face for fatigue detection. Therefore, the improved version of RetinaFace subtracts the regression prediction results of the face key points, significantly reducing the computation and improving the detection rate.

The three adequate feature layers obtained are equivalent to dividing the input image into grids of different sizes, and there are several a priori frames at each grid point; RetinaFace defaults to two a priori frames, and prediction results determine whether the a priori frames contain faces or not, and if they do, then the a priori frames are adjusted to obtain the prediction frames. Class Head determines whether the a priori frame at each grid point contains a face. Using 1 × 1 convolution, the number of channels in the feature layer is adjusted to num_anchors × 2; num_anchors is the number of a priori frames, which is defaulted to two in the RetinaFace network. Two is used to judge whether the a priori frame contains a face or not; two inside the serial number of one is more significant, as it means that the a priori frame contains a face. Two inside the serial number zero is more significant, meaning Box Head is used to adjust the center, width, and height of the a priori box to obtain the prediction box. Using 1 × 1 convolution, the number of channels of the feature layer is adjusted to num_anchors × 4, which is used to represent the adjustment parameters of each a priori box. Four represents the four adjustment parameters of the a priori box, the first two are used to adjust the center of the a priori box to obtain the center of the prediction box. The last two are used to adjust the width and height of the a priori box to obtain the width and height of the final prediction box. The process of actual frames can be divided into three steps:

(1) Calculate the degree of overlap between all actual frames and a priori frames, and use the a priori frames with IoU greater than 0.35 for prediction to obtain real frames;
(2) Coding these a priori frames that have a greater degree of overlap with the proper frame;
(3) The coding operation can be divided into classification prediction results and regression prediction results of the boxes.

**Figure 12:**
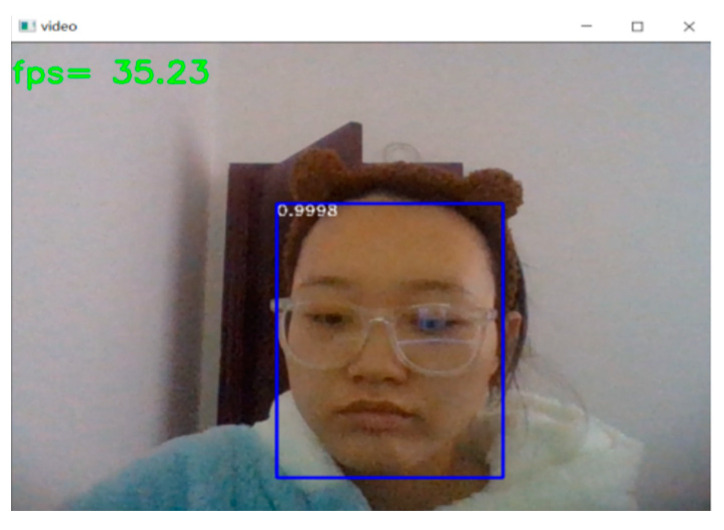
Face detection.

The improved algorithm reduces the recognition process of face feature points in the face detection part; firstly, the face is detected and boxed out using the RetinaFace algorithm, and then combined with the Dlib algorithm to detect 68 feature points on the driver’s face, which not only better accomplishes the recognition of 68 key points on the driver’s face, but also improves the rate of the RetinaFace face recognition algorithm. Figure 12 below shows the partial results of face detection after the improvement; five points are recognized before the improvement of the face feature point, and after the improvement of the feature point, this prediction step is removed.

**Figure 13:**
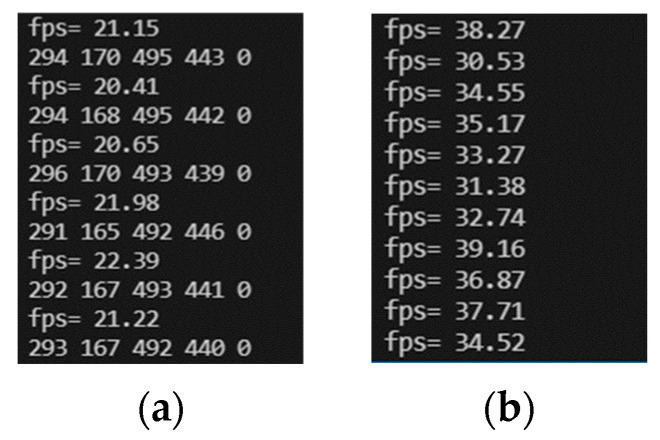
(**a**) Pre-optimization detection rate and (**b**) optimized detection rate.

Compared to the pre-improved network, the improved face detection rate is greatly improved with guaranteed accuracy, with fps going from an average of 16 to an average of 30, as shown in Figure 13.

**Figure 14:**
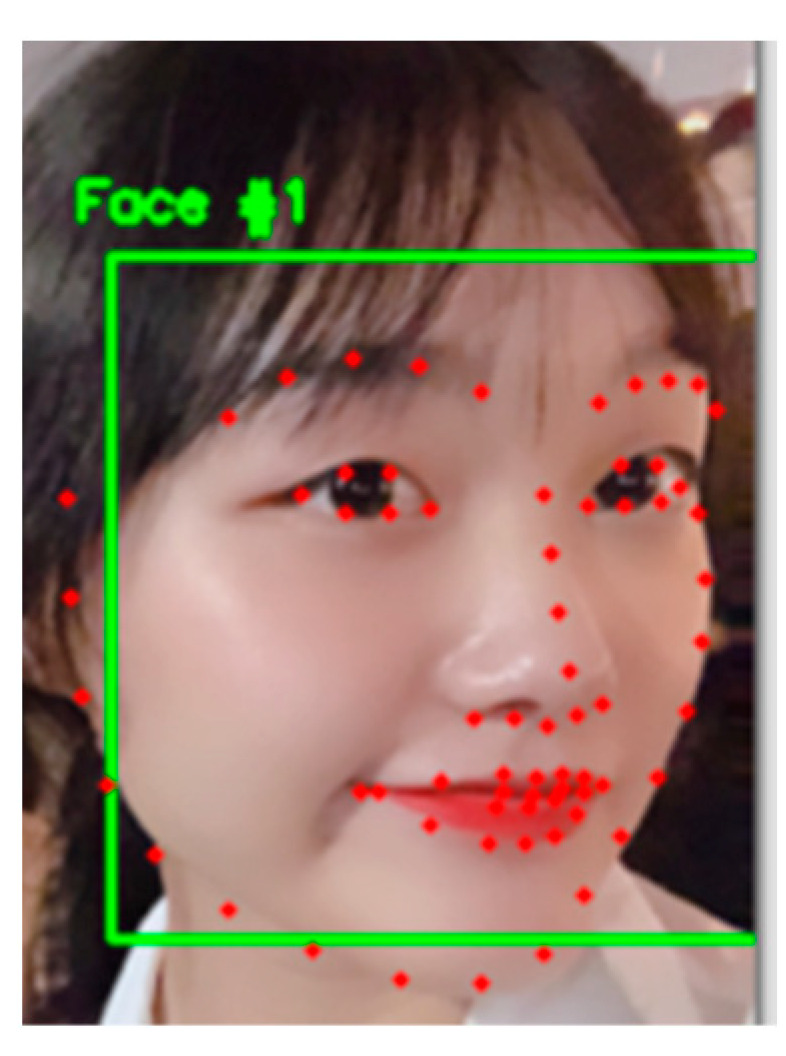
Keypoint detection.

**Figure 15:**
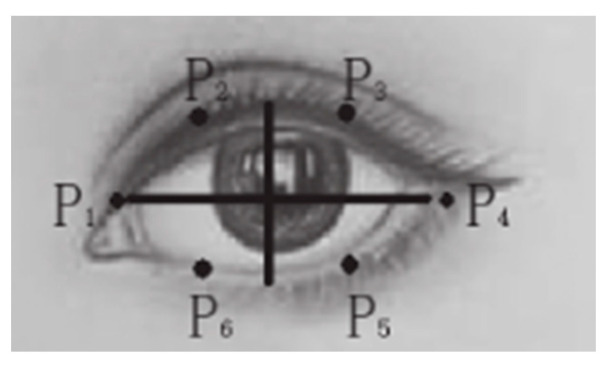
Eye feature points.

The 68 critical points of the driver’s face are detected by the Dlib algorithm, as shown in Figure 14. The opening and closing of the driver’s eyes are determined by the eye aspect ratio (EAR), as shown in Figure 15; P_1_–P_6_ are the six feature points of the eyes.

When the driver opens or closes his eyes, the aspect ratio will change. The formula of *EAR* is

(3) $$EAR=\frac{\left\|P_{2}-P_{6}\right\|+\left\|P_{3}-P_{5}\right\|}{2\left\|P_{1}-P_{4}\right\|}$$

**Figure 16:**
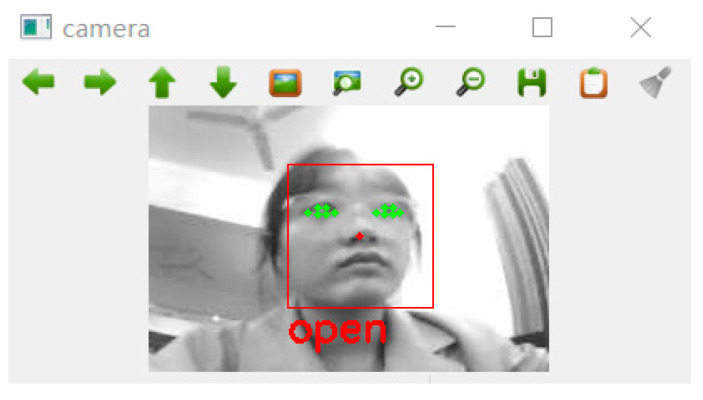
Eyes open.

The threshold for eye fatigue is set to 0.28; if the *EAR* is more than this, the eyes are considered open; if the *EAR* is less than it, the eyes are considered closed, as shown in Figure 16.

Combined with the *PERCLOS* (Percentage of Eyelid Closure over the Pupil) value, the fatigue state of the driver is detected, and if Equation (3) is satisfied, it is considered that fatigue driving is performed.

(4) $$PERCLOS=\frac{F}{T}\times 100\%$$

The *F* in Formula (4) represents eye closure frames, and the *T* in Formula (4) represents the total number of frames in the detection period.

**Figure 17:**
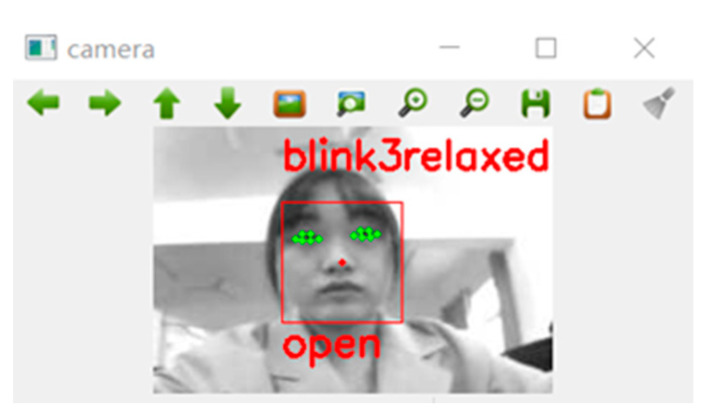
Driver status.

Referring to the relevant data [24,25,26], the fatigue limit is *PERCLOS* = 20%; we use 100 frames of images as a loop. If 20 frames of closed-eye images are detected in 100 frames, it is considered fatigued and the output is tired; otherwise, the output is relaxed, as shown in Figure 17.

**Figure 18:**
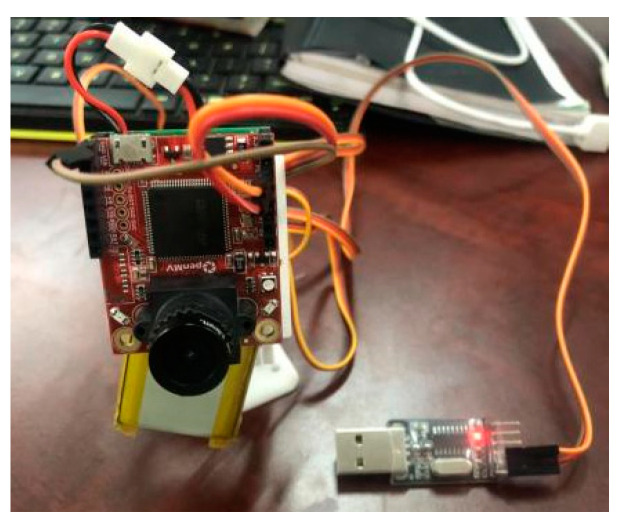
OpenMV Connection Diagram.

In order to test the effectiveness of the driver fatigue tracking detection algorithm in this paper and the influence of the dynamic tracking system after adding the gimbal to the driver fatigue detection effect. This paper conducts eIn order to ensure that the driver’s face is always within the monitoring range of the camera, ensure real-time and accurate detection, and avoid false detection caused by the driver’s face not being in the detection frame; the cradle head tracking system is added, as shown in Figure 18. The tracking gimbal of OpenMV first obtains the x and y coordinates of the center of the face, sends the coordinate information of the center point to OpenMV, and then controls the motion of the two servos of the gimbal by calculating the deviation between the coordinates of the center of the face and the center of the picture, to realize driving—personnel face tracking detection.

**Figure 19:**
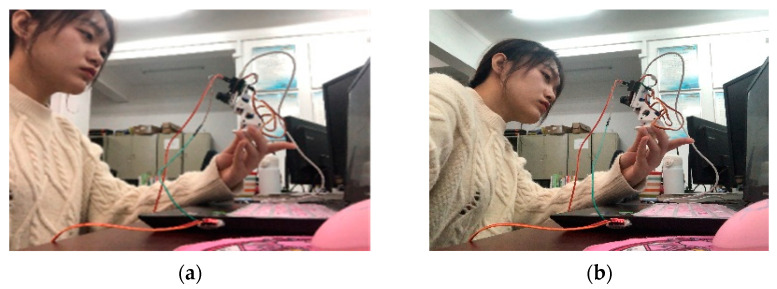
(**a**) Dynamic tracking of the detection process 1 (**b**) Dynamic tracking of the detection process 2.

In order to test the effectiveness of the driver fatigue tracking detection algorithm, and the influence of the dynamic tracking system after adding the cradle head tracking system to the driver fatigue detection effect, this paper conducts experiments on the subjects. This is done by testing whether the system recognizes and detects when the subjects are relaxed and fatigued using the driver fatigue detection system without the cradle head tracking system and the driver fatigue detection system with the cradle head tracking system, as shown in Figure 19.

Two kinds of driver fatigue detection systems without cradle head tracking system and cradle head tracking system are tested, respectively, and the two states of driver’s relaxation and fatigue are detected, respectively. Two thousand four hundred frames of images are identified and detected for each state. The detection results are shown in Table 3 and Table 4. The exact number of detections is used to ensure the effectiveness of the accuracy comparison.

The experimental results show that the detection accuracy of the driver’s relaxed state has increased from 92.5% when the gimbal is never added to 95% when the gimbal is added. The accuracy of the fatigue state has also increased to 94.17%, compared with that when the cradle head tracking system is not added. The accuracy of fatigue detection results is significantly improved, and dynamic tracking fatigue detection achieves better results.

## 5. Design of The Diver’s State Risk Level Discrimination System

This paper realizes the design of the driver’s state risk level discrimination system by integrating the driver’s fatigue detection and the detection of several dangerous driving behaviors. Through years of driving experience and analysis of related research, we found that fatigue driving is the most influential factor in car driving [27,28]. When driving, if the driver is drowsy, it will confuse the brain, and the vision will also become blurred, not to mention the fatigue of driving for a long time. Therefore, when performing risk classification, if there is fatigue driving, it is high-risk driving. We conducted a risk-level discrimination experiment on three behaviors of smoking, drinking, and making a phone call in the laboratory (non-driving environment). The experimental results show that when a person concentrates on one thing and suddenly receives a call, his attention will be diverted immediately. He will even drop his job if he is on the phone with someone important; if on the phone and he learns something that breaks him down, he can become out of control, let alone get on with the job at hand. Simultaneously, when answering the phone, you have to look at the mobile phone screen and tap the screen with your hand for a long time, which will affect the driver’s driving concentration. The reflection of the mobile phone will also interfere with the driver, making the driver unable to concentrate, resulting in traffic accidents. Therefore, we regard answering the phone as one of the most dangerous behaviors when driving, except for fatigue driving [29,30,31]. As far as smoking and drinking water are concerned, our lived experience tells us that drinking water has a more significant impact on driving because the cup is larger than the cigarette, it is easy to affect the driver’s vision, and in some cases, the driver must raise his head when drinking water, which will significantly affect the driver’s attention. Based on the above analysis, we rank the risk levels of the driver’s unsafe state behaviors studied in this paper (starting from the most dangerous) as follows: fatigue driving, making phone calls, drinking water, and smoking.

We combine the above four behavior state information and the 12-frame voting queue to determine the driver’s state danger level. We divide the driver’s dangerous state into three dangerous states: R1, R2, and R3. R1 represents very dangerous; we are continuously alert this situation and advise to stop driving; R2 stands for medium danger; we issue a danger warning to remind the driver to modify the current state of dangerous behavior in time, and continue to drive after correcting; and R3 represents a low-risk state, no alarm is required, and only the alarm flashing light needs to appear. The status level of the driver is queried according to Table 5 below.

In the experimental part, this paper collected 6000 frames of driver driving images, including 1920 frames of R1 risk level video, 2290 frames of R2 risk level video, and 1790 frames of R3 risk level video. The test results are shown in Table 6.

From the detection results of the driver’s driving state risk level, it can be concluded that the driver’s driving state risk level (R1, R2, and R3) has an accuracy rate of more than 90%. The accuracy rate of R3 hazard level detection even reached 96.3%, with a high accuracy rate. In experimental testing, we obtain better results, achieve the expected goals, and meet our needs for driving safety in real life.

## 6. Conclusions

In this paper, the YOLOX algorithm is used to detect specific target objects (cigarettes, water cups, and telephones) in the driver’s cab, and combined with the OpenPose posture detection algorithm to judge the driver’s dangerous behavior, and the accuracy of the detection results all reach 95%. The improved RetinaFace face detection algorithm is proposed, combined with the Dlib algorithm to determine the driver fatigue driving state according to the *PERCLOS* value, and the gimbal system is added to realize the dynamic tracking detection of the driver. A driver state danger level discrimination system is designed to classify four dangerous driving behavior states, namely, fatigue driving, drinking water, smoking, and making phone calls, and the experimental results show that the accuracy of R1, R2, and R3 danger level detection reaches 95.8%, 94.5%, and 96.3%, respectively.

With the development of deep learning and neural networks, the application in various fields is more and more comprehensive, the research scope and research data of this paper still need to be standardized and improved. In this paper, we only recognize the target objects (cigarettes, water cups, and telephones) in the driver’s cab, and the target objects are still relatively single, lacking the dataset of scenes in low-light and harsh environments, and insufficient for real-vehicle driving tests. Next, we will increase the variety of scenes and target objects in complex environments to make the dataset richer to test the robustness of the algorithm, modify the original information fusion structure of the network to enhance the feature fusion ability, modify the detection layer of the original network to enhance the ability of the network to extract and localize the effective information, and conduct a large number of practical scenes to test the discriminative system.

## Figures and Tables

**Table 1:** Test results.

	Recall Rate	Accuracy Rate
smoke	98.2%	97.8%
drink	98.5%	97.0%
phone	97.8%	96.6%
average	98.2%	97.1%

**Table 2:** Driver risky behaviors voting detection.

Risky Behavior Classification	No Voting Queue. (Accuracy Rate)	There is a Voting Queue. (Accuracy Rate)
Drink water	190/200 = 0.95	196/200 = 0.98
Smokes	186/200 = 0.93	190/200 = 0.95
Call up	188/200 = 0.94	196/200 = 0.98

**Table 3:** Fatigue test results without cradle head tracking system.

State	Test Numbers	Error Numbers	Accuracy
Relax	2400	180	92.50
Fatigue	2400	210	91.25

**Table 4:** Fatigue detection results when adding cradle head tracking system.

State	Test Numbers	Error Numbers	Accuracy
Relax	2400	120	95.00
Fatigue	2400	140	94.17

**Table 5:** Driver status level.

Action State Type	Queue Frames < 8	8 < Queue Frames< 16	16 < Queue Frames
call	R2	R2	R1
drink	R3	R2	R1
smoke	R3	R2	R2

**Table 6:** Danger level detection results of driving state.

Driver’s Driving State Danger Level	Number to be Checked	False Detection Rate	Detection Accuracy
R1	1920	81	95.8%
R2	2290	125	94.5%
R3	1790	66	96.3%

## Data Availability

The dataset is not readily available to the public as it is privately collected and produced.
